# Evolution of Sensor Research for Clarifying the Dynamics and Properties of Future Directions

**DOI:** 10.3390/s22239419

**Published:** 2022-12-02

**Authors:** Mario Coccia, Saeed Roshani, Melika Mosleh

**Affiliations:** 1 Department of Social Sciences and Humanities, CNR—National Research Council of Italy, 10135 Torino, Italy; 2 Department of Technology and Entrepreneurship Management, Faculty of Management and Accounting, Allameh Tabataba’i University, Tehran 1489684511, Iran; 3 Birmingham Business School, College of Social Sciences, University of Birmingham, Birmingham B15 2SQ, UK

**Keywords:** sensor research, sensor technology, network analysis, technological trajectories, technological change, scientific change, scientific development, science dynamics, industrial change, innovation management, wireless sensor networks, fiber-optic sensors, biosensors

## Abstract

The principal goal of this study is to analyze the evolution of sensor research and technologies from 1990 to 2020 to clarify outlook and future directions. This paper applies network analysis to a large dataset of publications concerning sensor research covering a 30-year period. Results show that the evolution of sensors is based on growing scientific interactions within networks, between different research fields that generate co-evolutionary pathways directed to develop general-purpose and/or specialized technologies, such as wireless sensors, biosensors, fiber-optic, and optical sensors, having manifold applications in industries. These results show new directions of sensor research that can drive R&D investments toward promising technological trajectories of sensors, exhibiting a high potential of growth to support scientific, technological, industrial, and socioeconomic development.

## 1. Introduction

The research field of sensors is undergoing a significant change to support the evolution of science and technology in society [1,2]. The goal of this study is to analyze the scientific ecosystem of sensor research (networks incorporating manifold research fields) over time to show science dynamics supporting technological trajectories directed to fulfil human goals and needs and to solve problems in society. In particular, this paper investigates sensor research from 1990 to 2020 to clarify changes in the scientific structure (network) of research fields and technologies over these 30 years [3,4,5,6]. In general, scientific change in sensors supports the evolution of technologies as well as industrial and social change, such as smart or intelligent sensors [7,8,9,10] and the Internet of Things [11,12,13,14]. The study here endeavors to explain the dynamics of sensor research using networks or maps of scientific publications over time that serve as a main unit of analysis to understand the organization and evolution of science and technology [15,16,17]. The crux of this study is rooted in scientometrics (the study of quantitative characteristics of science and scientific research), and given that this approach is uncommon in this journal that focuses on the technical and engineering aspects of sensors, a brief background is useful to understand and clarify it.

Leydesdorff (2007) developed maps of journals, showing centrality measures to clarify citation environments (small sets of journals where citing is above a certain threshold) [18]. Another approach by Klavans and Boyack (2006) identified a new measure of relatedness among bibliometric units (e.g., journals, words, etc.) for mapping science and providing critical aspects for the structure and evolution of science [19]. Relatedness measures also have a vital role in showing the relationship among data items [20]. Small (1999) argued that the network of linkages from document to document and from discipline to discipline can show crossover fields and offer the possibility of exploring extended knowledge pathways and new technological trajectories [21]. Boyack et al. (2005) maintained that science maps provide main aspects to analyze fields of research and emerging technologies, and their interconnectedness [22]. Scholars also argued that emerging general-purpose technologies and new discoveries induce radical novelty, accelerated growth, and main socioeconomic impacts [23,24,25,26,27]. Manifold techniques have been developed in scientometrics and studies of innovation to detect and analyze the evolution of research fields and technologies [16,28,29,30,31,32,33,34,35]. These methods are based on large datasets and computational approaches that allow the computing of specific indicators for detecting patterns in science and new pathways of technological trajectories [36]. Quantitative approaches, based on bibliometric data of publications, are useful techniques to capture information earlier in the cycle of technology development, whereas patents, in contrast, trail behind [37]. In this research stream, the study presented here has the purpose of mapping the scientific ecosystems of sensor research (ecosystem here is a community of research fields and technologies that interact and evolve over time), on the basis of publications, to analyze the evolution of science and technology in sensors from 1990 to 2020. This technology analysis, based on updated data, clarifies the interactions among research fields and technologies that sustain the evolution of promising sensors directed to next industrial and economic change. Overall, the explanation here of dynamic relationships of research fields within sensor networks over time can drive R&D investments and management of technology to foster the scientific and technological development of sensors towards new directions having fruitful applications in manifold industries.

## 2. Materials and Methods

### 2.1. Data Processing Resources

In this study, we used the Web of Science (WOS) Core collection database 2022 to retrieve sensor research and technology literature documentations [38].

Web of Science is a main source of data for bibliometric analysis of sensor research [38]. Web of Science includes approximately more than 10,000 journals [39]. Web of Science is used here because it offers a large amount of scientific information and a variety of metadata, including abstracts, institutions, citations, etc., which are crucial aspects for accurate bibliometric analysis of science and technological evolution [40,41,42].

The term “sensor” was searched in Web of Science Core Collection (2022) in the section of topics [38]. The results were refined by document type = (Articles), Language = (English), Publication years = 1990–2020, and Indexes = (SCI-EXPANDED).

The sample contained 362,745 papers split into three distinguished timespans, given by 1990–2000, 2001–2010, and 2011–2020.

### 2.2. Data Processing Procedure and Computational Approach for Network Analysis

To address the main purpose of this study, we used articles original keywords (DEs) as the basis for building the keywords co-occurrence in networks regarding sensor research and technologies. We also implemented this approach to visualize the interconnection among sensor research fields and technologies to analyze and interpret the evolving relationship among in sensor networks. We also used the co-occurrence measurement to study the interconnection among different sensor sub-technologies [43]. The methodology of co-occurrences is commonly used for identifying the underlying collaborative structure among terms. Two terms (keywords, journals, research disciplines, countries, authors, etc.) co-occur whenever they simultaneously appear in a single document [44]. Scholars have widely used this approach to analyze the interconnection among different research fields [45]. In this study, we used the “Original Keywords” as the basis for representing the sensor research and technologies and creating the interconnection network among words. These words are known by the DE tag in the Web of Science bibliometric data, and they are separated by a semicolon. In particular, the construction of co-occurrence networks among words is based on following data processing procedure:

▪ Bibliographic data were downloaded from the Web of Science (2022) database [38] and split into three periods: 1990 to 2000, 2001 to 2010, and 2011 to 2020.
▪ All the combined phrases that lacked “sensor”, “sensing”, or “sense” and adjective clauses were removed. This step focused only on words related to sensor technologies (for instance, biosensors, wireless sensor networks, gas sensors, etc.)
▪ We used Python programming language version 3.6.5 [46] and Scikit-learn library version 0.23.2 for constructing the co-occurrence matrix [47]. In this step, we determined a threshold and removed the words with fewer than ten co-occurrences.
▪ Afterwards, we utilized Gephi software version 0.9.2 to visualize the matrix of co-occurrences and calculate the network measures [48]. The node indicates the words related to sensor research and technologies, and a link makes a connection between two words whenever they appeared in at least ten articles. To put it differently, a link means two different words co-occurred in at least ten articles. The color of nodes represents the community: when two nodes have a similar color, they are in the same community in the classification. The thickness of each edge represents the weight of co-occurrences. If more than two terms appeared in the same documents; the connected edge will be thicker.

After creating the networks of word co-occurrences for each period, we applied measures to analyze the structure and explain the evolving pathways of sensor research and technologies over time [49]. In particular, measures are:

− Degree centrality (*DC*) indicates the number of edges a node has [50]. In the word co-occurrence networks, degree denotes the total number of words that appear with the node in the same documents. Degree centrality of node *v* is given by:

$$DC\left(v\right)=\sum_{v,g=1}^{n}Edge\;\left(g\right)$$

where

*DC*(*v*) = degree centrality of node *v*

*g* = edge

− Betweenness centrality (*BC*) indicates how essential a node is to create connections with other nodes in the shortest path. Betweenness centrality of node *v* is calculated by following formula [51,52]:

(1) $$BC\left(v\right)=\sum_{s\neq v\neq t}\frac{\sigma_{st}\left(v\right)}{\sigma_{st}}$$

where

*BC* = betweenness centrality measure of node *v*

*σ_st_* = total number of shortest paths from nodes *s* to node *t*

*σ_st_* (*v*) = number of shortest paths from *s* to *t* going through *v*

− A node’s closeness centrality (*CC*) is an indicator of a network centrality: it is the number of links needed to connect each node in the network with all the other nodes in the network or the average number of links required to reach all other nodes in the network from a node in the network [6].

$$CC\left(v\right)=\frac{1}{\sum_{u\in V}d\left(v,u\right)}$$

where

*CC* = closeness centrality measure of node *v*

*d*(*v*, *u*) is a shortest path between nodes *v* and *u*

∑ is the sum of the path lengths from node *v* to all other nodes in the network

− Finally, community structure represents the categorization of technologies interconnection using the modularity algorithm to distinguish the classifications [53]. The number of communities calculated by modularity function (*Q*) is:

(2) $$Q=\frac{1}{2m}\;\sum_{i,j}\left[\;A_{ij}-\frac{k_{i}k_{j}}{2m}\right]\delta \left(c_{i},c_{j}\right)$$

where

*Q* = modularity function

$A_{ij}$ = weight of the connection from i to j

$k_{i}\;degree\;of\;vertex\;i$ = $\sum_{j}A_{ij}$ = sum of the weights of the edge attached to vertex *i*

$k_{j}$*degree of vertexj* = $\sum_{i}A_{ij}$ = sum of the weights of the edge attached to vertex *j*

$c_{i}\;$ = community to which vertex *i* is assigned

$c_{j}$ = community to which vertex *j* is assigned

*m* = ½ ∑*ij* $A_{ij}$ = number of edges in the graph

$\delta -\mathrm{function}\;\delta \left(c_{i},c_{j}\right)$ is 1 if $c_{i}$ *=* $c_{j}$ and 0 otherwise.

Networks of co-occurrence generated by Gephi were saved in GraphMl format and imported into SCI2 software version 1.3 to implement the community detection algorithm.

We used degree centrality (*DC*) to analyze the evolution of nodes over time and utilized community structuring to detect the classified technologies that had the highest interconnections to track the transition of linkages between sensor research and technologies. We also used betweenness centrality (*BC*) measures to indicate the nodes’ role in facilitating the connection of sub-technologies at the heart of three networks. Nodes with the highest score of BC are positioned to be a bridge for connections among the other network nodes [54].

## 3. Results and Discussion

### 3.1. The Ecosystem of Sensor Research and Technologies in the 1990–2000 Period

**Figure 1:**
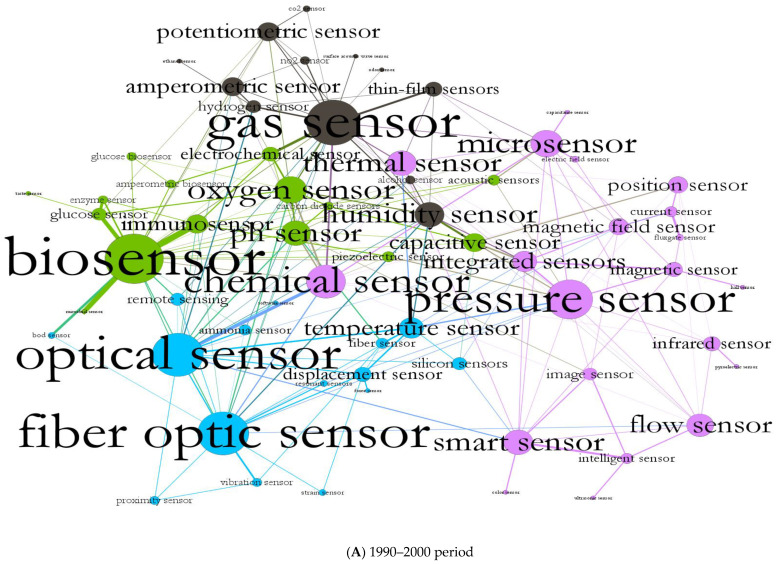

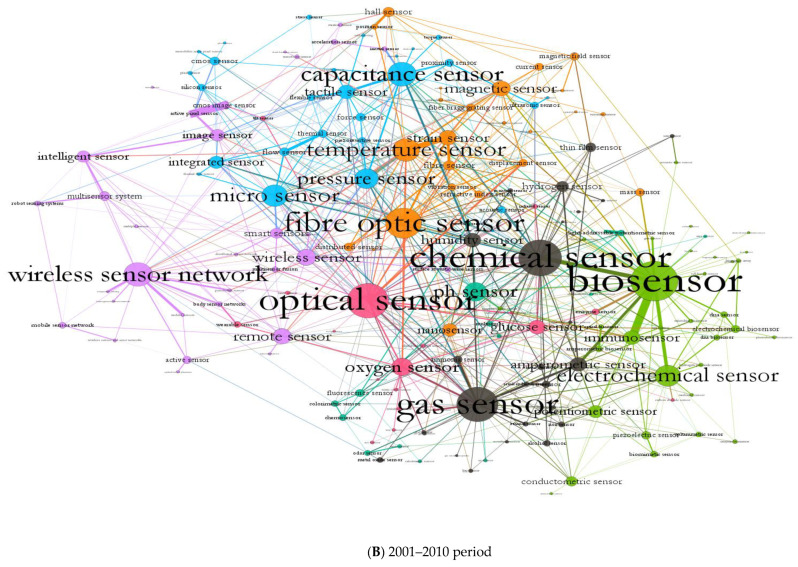

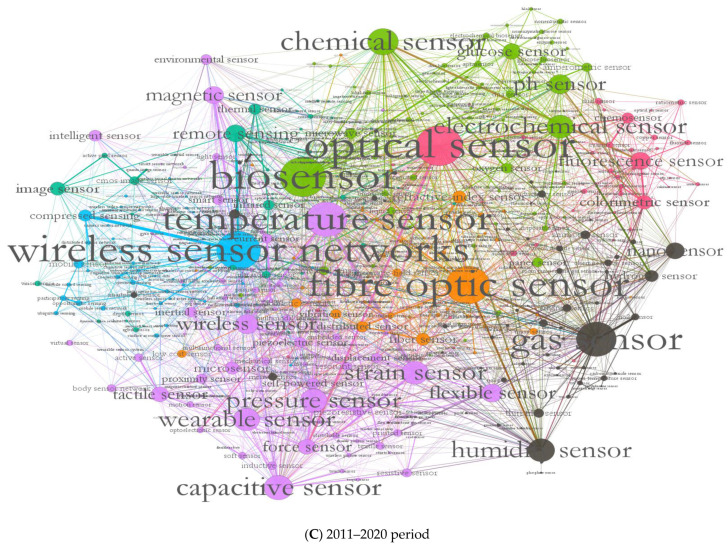
Structure and evolution of scientific and technological networks in sensors over time (**A**) from 1990 to 2000. (**B**) from 2001 to 2010. (**C**) from 2011 to 2020.

The ecosystem of sensor research and technologies in the 1990–2000 period shows a network described in Figure 1A. The total number of articles in this dataset is 30,674 records, 8.45 percent of the total articles. Figure 1A also shows the network of co-occurrences of these terms from 1990 to 2000. Figure 1A (1990–2000) includes 72 nodes, 194 edges and 5 communities. Table 1 shows that “biosensor”, “gas sensor”, and “optical sensor” have the highest degree centrality compared with other nodes: these three technologies have a higher interaction with other technologies. Results of Table 1 also suggest a high centrality degree for “fiber optic sensor” and “pressure sensor” among all nodes in the network. There are four communities in Figure 1A, in which “biosensor”, with a centrality degree score of 23, has a strong relationship with “oxygen sensor”, “ph. sensor”, “immune sensor”, and “capacitive sensor”. Based on edge weight, these technologies have a high level of co-occurrence in documents leading to an interconnected community. Moreover, “gas sensor”, with a centrality degree score of 21, is in the head of community 4, strongly connected to other sub-technologies, including “humidity sensor”, “potentiometric sensor”, and “amperometric sensor”. In the second community, the “optical sensor”, with a centrality degree score of 20,is highly connected to the “fiber optic sensor”, “temperature sensor”, and “displacement sensor”. The remaining technologies are classified in community 1, which has the highest number of nodes. In this community, the “pressure sensor”, with a degree number of 18, is highly interconnected with “chemical sensor”, “micro sensor”, “smart sensor”, “thermal sensor”, and “integrated sensor”.

### 3.2. The Ecosystem of Sensor Research and Technologies in the 2001–2010 Period

This period shows an ecosystem based on a network with a growing number of nodes (197) and edges (623). This period contains 83,512 records, 23.02 percent of the total articles. This period has nine communities. Figure 1B shows that the leading technologies in the ecosystem of the 2001–2010 period are “biosensor”, “chemical sensor”, “gas sensor”, and “optical sensor”. The most interconnected technologies, considering the edge weight, are “active pixel sensor” with “CMOS image sensor”, “biosensor” with “immunosensor”, “strain sensor” with “temperature sensor” and “biosensor” with “chemical sensor”. Table 1 shows that the top five sensor technologies in the 2001–2010 period are “biosensor”, with centrality degree of 53, included in community 2, which is highly connected to “electrochemical sensor”; “chemical sensor”, with centrality degree of 48, is included in the fourth community with “gas sensor”, having centrality degree of 46, and with “humidity sensor”. “Optical sensor”, with centrality degree of 46, is highly connected with “oxygen sensor” and “glucose sensor” as community 6. This result confirms the growing role of optical sensors as forecasted by Andersen et al. [1]. Moreover, “fiber optic sensor”, with centrality degree of 40, has the highest interconnection with “temperature sensor”, “magnetic sensor”, and “strain sensor” and is included in the fifth community. These nodes, with the highest level of centrality degree score among all the nodes, represent a highly diversified interconnection with other sensor technologies compared with other nodes in this network. Interestingly, unlike the previous period, the “fiber optic sensor” is a separate element that acts as a new interconnected network from the “optical sensor” community. Our results show that during the second decade, the “wireless sensor network”, “wireless sensor”, and “remote sensor” co-occurrences in documents gained momentum with other technologies and emerged in the top 20 topics with the highest level of degree centrality.

### 3.3. The Ecosystem of Sensor Research and Technologies in the 2011–2020 Period

Finally, the ecosystem based on interconnection between sensor research and technologies in 2011–2020 contains 248,559 records, 68.53 percent of all articles collected in this study (see Figure 1C). The co-occurrence network of sensor technologies comprises 553 nodes and 2696 edges. “Strain sensor” with “temperature sensor”, “compressed sensing” with “wireless sensor network”, “biosensor” with “immunosensor”, “rechargeable sensor networks” with “wireless sensor network”, and “colorimetric chemosensor” with “fluorescent chemosensor” have strong relationships based on their edge weight score and are classified in eight communities [55,56,57]. Figure 1C shows that the size of nodes and network linkage have been growing and creating a large and intense ecosystem on the basis of complex interconnection communities among manifold sensor research fields and technologies. The leading technologies in this period are “optical sensor”, with a centrality degree of 128, “biosensor”, with a centrality degree score of 126, “wireless sensor network”, with a centrality degree score of 121, “fiber optic sensor”, with a centrality degree of 120, and “temperature sensor”, with a centrality degree of 111. Table 1 shows the top 20 technologies considering centrality degree values. The top five sensor technologies are “optical sensor”, with a centrality degree score of 128, included in community 6, which has a high interaction with “fluorescent sensor”; “biosensor”, with a centrality degree of 126, is strongly associated with “chemical sensor” and “electrochemical sensor “. Surprisingly, these two technologies with the highest centrality degree in the previous decade have their separated interconnection in two different communities with other technologies. In this decade, although the “chemical sensor” rank based on the degree of centrality decreased, it began a process of merging with “biosensor” in the same community. The growing role of biosensor in ecosystem confirms the preliminary study by Andersen et al. (2004), where the evolution and potential aspects of this sensor are rather ambiguous [1]. The “wireless sensor network”, with a centrality degree of 121, expands its interconnection community and obtains the third rank in a degree centrality scoring. Moreover, sensor technologies in community 3 are not present in the top 20 topics; this vital finding suggests that although the “wireless sensor” technology increases its interconnection and diversification with other related technologies, it is an emerging technology that has not generated strong relationships with other top technologies. This evolutionary characteristic is also present in “fiber optic sensor”; moreover, the “optical sensor”, that was the head of the community including “fiber optic sensor”, has stopped its growth and is not in the top 20 topics having high degree centrality score. Instead, the “temperature sensor”, which was included in the same community with “fiber optic sensor”, emerged as a new community in this decade and started expanding its own technology interconnection community.

### 3.4. General Discussion of the Evolution of Sensors, 1990–2020 Period

The evolution of the scientific ecosystem of sensors is represented here with a change of network indicators. In particular, the network’s average degree increased from 5.4 to 10.6 during the 1990–2020 period and suggests growing interconnections. However, the density of interconnection within structures has been decreasing from the first decade: the degree of closeness centrality in networks and the interconnection among their elements based on their co-occurrence have deteriorated. Moreover, the decreasing magnitude of betweenness centrality demonstrates that there is a lower dependency on some nodes having a bridging role, such that many sensor technologies have connections directly with other technologies instead of making connections through intermediate/bridge technologies. Results also show the increasing level of closeness centrality and the stable number of communities in networks [52]: technological interconnection in sensors tends to be more centralized, and the differences among communities tend to gradually increase. Results also show that the top ten units in sensors have an evolution from three perspectives: direct connection, interconnection, and diversified interconnection. The centrality degree of a single node in a network indicates the potential aspect that could facilitate the interaction within the network [43]. In addition, results reveal that optical sensor, biosensor, fiber optic sensor, and wireless sensor are central technologies directly linked to other nodes in the network [57,58,59,60,61]. The wearable sensor, which has emerged later than other technologies, tends to have a high potential growth and interaction with other sensor technologies (measured with rapid improvement in degree of centrality). Finally, sensor technologies tend to have a greater capacity to interact with other technologies [62]; this finding is consistent with the theory of technological parasitism by Coccia [63,64,65].

The technologies with a higher closeness centrality score have a low distance from their community nodes and a high distance from other excluded nodes. The technologies with a high level of closeness centrality (*CC*), including “optical sensor”, “biosensor”, “fiber optic sensor”, “gas sensor”, and “wireless sensor networks”, have a powerful evolution and create distinct communities. Aside from *DC* and *CC* score, the top ten technologies with higher betweenness centrality have a higher diversification than technologies with the highest level of closeness centrality.

The evolution of interconnection among items in the top 20 sensors from 1990 to 2020 is in Table 2. Sensors with the highest centrality degree scores in 1990–2000 (biosensor, gas sensor, optical sensor, fiber optical sensor, pressure sensor, and chemical sensor) have been expanding over time. Some technologies, such as “strain sensor” had a low centrality degree score in the first period, but it increased to 18 and ranked 17 in the second period, reaching, consequently, centrality degree score achieves the level of 70 and rank 10 in the last period (2011–2020). Moreover, the rank of “temperature sensor” improved from 15—with an initial degree of 9 (first period), rank 8 and a degree score of 29 in the second period—to rank 5 with a degree score of 118 in the last period under study. The “capacitive sensor” ranked initially 18 and elevated ultimately to 8 in the last period, whereas “electrochemical sensor”, with a degree of 6 and rank 23, improved to rank 13 with a degree score of 69 in the last period.

These results show that technological positions have evolutionary phases of transition in the network and converge towards vital nodes with growing levels of interconnections over time [6,64,66,67].

Table 3 shows new items of sensors that emerged in the network after the 1990–2000 period and started an evolutionary growth of publications. In particular, new items about sensors increased from 137 in 2001–2010 to 374 in the 2011–2020 period. This finding reveals that sensor research has a significant and continuous evolution in science and technology.

Table 3 focuses on the top 20 items in sensor research that emerged in networks from 2001 to 2020, whereas Table 4 shows strictly those items that are associated with sensor technologies that have a high growth of publications and that have critical aspects for the development of sensors. This result demonstrates the significant and ongoing evolution of sensor research and technology.

### 3.5. Properties of the Evolution of Networks in Sensor Research

These results suggest some properties of the scientific change of the ecosystem of sensor research and technologies that can support general principles for the evolution of science and technology [6,26,28,32,68]:

Firstly, sensor research evolves in networks with complex interactions among different fields and technologies. In fact, the level of interconnections between sensor-related research and technologies is increasing over time dramatically.

Secondly, some sensor technologies achieve a critical position in the network, playing a connective role of master technology for other technologies. For instance, wireless sensor networks increased exponentially in the ecosystem, fulfilling a bridging and supporting role compared with other technologies.

Thirdly, sensor research is generating new trajectories of both general-purpose technologies and specialized technologies during their co-evolutionary pathways over time.

**Figure 2:**
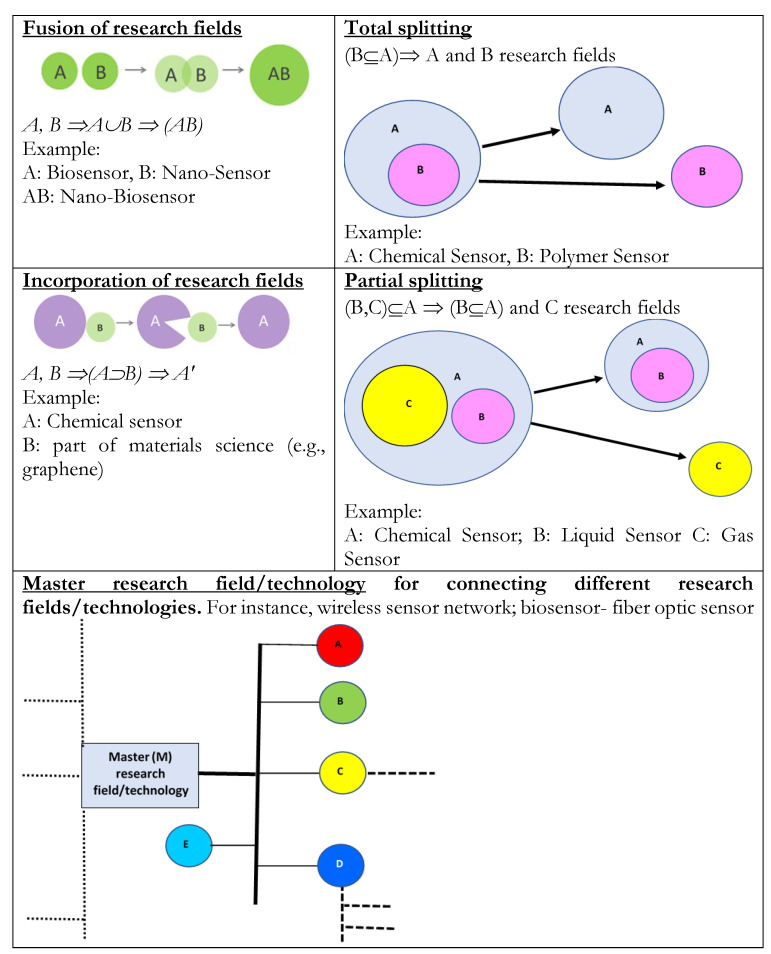
Patterns of evolution of science and technology based on characteristics of networks. *Note*: Capital letters (e.g., A, B, …, E) in small circumferences of figures above (using terminology of set theory) indicate research fields or technologies.

Comparative analysis of maps over time suggests also that the evolution of sensor research proceeds with the following typologies (Figure 2):

□ Total fusion of research fields is when two or more research fields (e.g., A and B) merge and create a new one (i.e., AB) that evolves as a whole system. For instance, in sensor research, nano-bio sensor is a fusion of nanosensor and biosensor. In particular, the combination of these two technologies and research fields created a new potential field.
□ Partial fusion is, during the scientific change, the incorporation of a smaller research field (e.g., B) into a large research field (e.g., A), generating a super research field A’ (that embodies B). For instance, in sensor research, the “chemical sensor” includes areas of materials science (e.g., graphene) with the goal of generating ion/molecule sensors applied in pharmaceutical and food production.
□ Total splitting (total fission) is when a research field A (including a sub-research field B) splits into research fields A and B that have autonomous evolutionary trajectories. For instance, in sensor research, polymer sensor is a technology born in the chemical sensor community, which then grew up independently and created its own domain of study and evolutionary pathway.
□ Partial splitting (partial fission) is when research field A (containing sub-research fields B and C) develops by splitting into a research field A’’, also containing B, and a research field C that splits off from the original set A; both research fields have autonomous evolutionary trajectories. For instance, in sensor research, both gas sensors and liquid sensors dawned in the chemical sensors field; eventually, gas sensors began their evolution independently from chemical sensors and created their own domain; however, liquid sensors still cannot be considered as a dependent province of science, and its expansion is intertwined with growth of chemical sensors.
□ Master technologies have a connective role for other technologies with an integrated-based structure by bridging and supporting the development of other inter-related technologies, such as wireless sensor networks, biosensors, and fiber optic sensors. They play a vital role in integrating elements of the networks and connecting sensor technologies to create new paths through evolution of science and technology. Master technologies increase exponentially in ecosystem of sensor research.

## 4. Conclusions, Limitations, and Prospects

The novelty of this study is the examination of the structure and dynamics of sensor research with networks that show interactions among research fields directed to support scientific and technological trajectories for development of science and technology in society. In particular, what sets this study apart from others is that we have used specific scientometric methods, based on publications from 1990 to 2020, in order to show interactions between research fields and technologies that explain the evolutionary paths of sensors over time. Results show that the evolution of sensor research over the last few decades is unparalleled [1]. Sensor technologies are co-evolving with growing interactions among fields of research and technologies directed to advance science and technology to fulfil human goals and needs and to solve problems in society [63]. For instance, the evolution of smart sensors is associated with the integration of the Internet of Things, through which it is possible to connect devices and exchange information among people and systems [69]. The characteristics of evolutionary pathways in sensor research, described here, can improve the allocation of R&D investments in private and public organizations for beneficial social impact [70]. Results reveal that the ecosystem of sensor research is rapidly growing from 2011 to 2020 with a dense network of interconnection [62,71]. In this period, more than 300 sensor units (research fields or technologies or new topics) emerged, developed, and connected to others, such as “biosensor”, “fiber optic sensor”, “wireless sensor network”, “gas sensor”, and “optical sensor. Moreover, results suggest that in the last decade, sensor technologies are moving towards pathways of specialization generated by a process of splitting from other technologies or large research fields. For example, gas sensors are generating pathways of specialization such as: “metal oxide gas sensor”, “optical gas sensor”, “electrochemical gas sensor”, “calorimetric gas sensor”, “acoustic-based gas sensor”, etc. The “smoke sensor”, “LPG sensor”, “carbon monoxide sensor”, “hydrogen sensor”, “ammonia sensor”, etc., are also the result of the development and specialization of gas sensor technologies. Consequently, industrial change and manufacturing systems will be directed to specialized applications of sensors. In fact, the stabilizing number of communities and the increasing level of closeness centrality in networks here indicate that sensor research evolves both with processes of specialization and of merging that capture complementary aspects of different technologies and research fields [63,64,66,72,73,74]. Hence, this study suggests that sensor research and technologies are in continuous evolution because of recent advances in different research fields, such as information and communication technologies, artificial intelligence, internet of things, nanoscience, etc. What this study adds, compared with other contributions, is synthetized in the following two points.

### 4.1. Contribution to Theory

The study suggests various theoretical properties that can clarify the evolution of science and technology in sensors:

- Sensor technologies evolve with increasing interactions among different research fields and innovations.
- Sensors evolve with technological trajectories directed to specialized innovations that solve problems.
- Sensor research evolves with processes of:
  − Total fusion of different inter-related research fields
  − Partial fusion with the incorporation of a smaller research field into a large research field
  − Total splitting (total fission) when a research field splits up in different research fields
  − Partial splitting (partial fission) when a research field develops by splitting part of its elements in a new research field having an autonomous trajectory of growth
  − Master technologies that have a connective role for other inter-related technologies, thus supporting a systemic evolution.

### 4.2. Management and Policy Contribution

Policymakers, managers, and scholars know that financial resources can be an accelerating factor of progress and diffusion of science and technology to support the scientific and technological development in society [17,70]. This study provides critical information with network analysis to guide innovation management to allocate economic resources with effectiveness towards research fields and technologies that have a growing centrality degree and specific levels of closeness and betweenness (e.g., wireless sensor networks) to foster the technological development for positive industrial and impact societal. In fact, these findings can support policymakers and funding agencies in making appropriate decisions regarding sponsoring specific research fields and technological trajectories in sensors that have a high potential growth and promising applications in industries with fruitful effects for the current and future economic and social change.

Although this study has provided some interesting, albeit preliminary results, it has several limitations that future research should reduce with new data and approaches to reinforce proposed results here.

### 4.3. Limitations

First, a limitation of this study is that sources under study may capture only certain aspects of the ongoing dynamics of sensor research. Second, there are multiple confounding factors that could have an important role in the evolution of sensor research to be further investigated in the future, such as discoveries, high R&D investments, collaboration intensity, openness, intellectual property rights, etc. Third, the computational and statistical analyses in this study focus on a specific period that can be extended in future investigations. Fourth, some technologies detected, such as oxygen sensor, can be an electrochemical sensor (device) or an optical device or a “gas sensor”. In order to identify the type of sensor (e.g., in oxygen sensor), it is necessary to analyze the contents of related articles. However, in the bibliometric approach applied here, the content of texts cannot be examined due to the structure of data and the large sample under study, which is one of the drawbacks of the method.

### 4.4. Future Research

Future development of this study should be directed to design new indices of technometrics on the basis of measures of betweenness, closeness, and degree centrality of networks to assess and predict the evolution of new technological trajectories in sensors, as well as to support implications of innovation management. An additional approach for future inquiries can be a content analysis to examine articles’ content and provide a coherent understanding of hidden patterns in unanalyzed texts as part of a systematic review of the literature in sensor research [75,76,77]. In short, the content analysis of articles and their systematic review with the PRISMA protocol can support complementary results to further explain the evolutionary dynamics of sensor research and technologies [78].

To conclude, the results presented here clearly illustrate the evolutionary paths of sensor research that are based increasingly on growing interactions among research fields and technologies directed to science advances and technological change for supporting industrial and socioeconomic development [79,80,81,82]. However, a continuing and detailed examination is needed for improving technological forecasting and supporting appropriate strategies of innovation management of sensor technologies directed to foster technological and economic change for better economies and societies.

## Figures and Tables

**Table 1:** Top 20 units of sensor in networks having the highest centrality degree during the 1990–2020 period.

1990–2000			2001–2010			2011–2020		
Word	Degree Centrality	Community	Word	Degree Centrality	Community	Word	Degree Centrality	Community
biosensor	23	3	biosensor	53	2	optical sensor	128	6
gas sensor	21	4	chemical sensor	48	4	biosensor	126	2
optical sensor	20	2	gas sensor	46	4	wireless sensor network	121	3
fiber optic sensor	20	2	optical sensor	46	6	fiber optic sensor	120	5
pressure sensor	18	1	fiber optic sensor	40	5	temperature sensor	111	1
chemical sensor	16	1	wireless sensor network	31	1	gas sensor	109	4
micro sensor	12	1	capacitive sensor	31	3	chemical sensor	83	2
oxygen sensor	12	3	temperature sensor	29	5	capacitive sensor	77	1
humidity sensor	11	4	micro sensor	28	3	pressure sensor	72	1
ph. sensor	11	3	electrochemical sensor	27	2	strain sensor	72	1
smart sensor	11	1	pressure sensor	25	3	humidity sensor	72	4
thermal sensor	11	1	ph. sensor	24	7	electrochemical sensor	71	2
flow sensor	10	1	oxygen sensor	22	6	wearable sensor	70	1
temperature sensor	10	2	wireless sensor	20	1	wireless sensor	59	1
integrated sensor	9	1	magnetic sensor	19	5	ph. sensor	59	2
immunosensor	9	3	remote sensor	19	1	flexible sensor	55	1
capacitive sensor	8	3	strain sensor	18	5	magnetic sensor	53	1
potentiometric sensor	8	4	glucose sensor	17	6	fluorescent sensor	52	6
amperometric sensor	8	4	humidity sensor	17	4	remote sensor	52	7
displacement sensor	7	2	amperometric sensor	17	3	nano sensor	49	4

*Note*: Some sensor units detected in this table can have different applications, such as oxygen sensor, which can be an electrochemical sensor (device), an optical device, or a “gas sensor”. In order to identify the type of sensor and its application (e.g., in oxygen sensor), it is necessary to analyze the contents of related articles. However, in the bibliometric approach applied here, the content of texts cannot be examined due to the structure of the data and the large sample under study.

**Table 2:** Top 20 items in sensor research with measures of networks from 1990 to 2020.

1990–2000					2001–2010					2011–2020				
Label	*DC*	*BC*	*CC*	Community	Label	*DC*	*BC*	*CC*	Community	Label	*DC*	*BC*	*CC*	Community
biosensor	23	0.149	0.563	2	biosensor	53	0.135	0.562	1	optical sensor	128	0.122	0.556	5
gas sensor	21	0.128	0.558	3	chemical sensor	48	0.080	0.555	3	biosensor	126	0.137	0.553	1
optical sensor	20	0.131	0.563	1	gas sensor	46	0.090	0.538	3	fiber optic sensor	120	0.126	0.544	4
fiber optic sensor	20	0.113	0.553	1	optical sensor	46	0.067	0.553	5	wireless sensor networks	118	0.146	0.532	2
pressure sensor	18	0.072	0.525	0	fiber optic sensor	40	0.072	0.525	4	temperature sensor	111	0.079	0.543	0
chemical sensor	15	0.042	0.534	0	wireless sensor network	31	0.056	0.488	0	gas sensor	109	0.095	0.535	3
microsensor	12	0.063	0.488	0	capacitive sensor	31	0.045	0.487	2	chemical sensor	83	0.044	0.515	1
oxygen sensor	12	0.032	0.496	2	temperature sensor	29	0.017	0.491	4	capacitive sensor	75	0.035	0.507	0
humidity sensor	11	0.018	0.473	3	micro sensor	28	0.026	0.517	2	strain sensor	70	0.032	0.506	0
ph. sensor	11	0.036	0.484	2	electrochemical sensor	27	0.028	0.482	1	pressure sensor	72	0.030	0.488	0
smart sensor	11	0.052	0.462	0	pressure sensor	25	0.013	0.472	2	humidity sensor	70	0.029	0.496	3
thermal sensor	11	0.014	0.469	0	ph. sensor	24	0.021	0.486	6	wearable sensor	70	0.034	0.511	0
flow sensor	10	0.032	0.480	0	oxygen sensor	22	0.030	0.478	5	electrochemical sensor	69	0.049	0.501	1
integrated sensors	9	0.020	0.473	0	wireless sensor	20	0.013	0.461	0	wireless sensor	59	0.025	0.484	0
temperature sensor	9	0.016	0.449	1	magnetic sensor	19	0.021	0.446	4	ph. sensor	59	0.024	0.498	1
amperometric sensor	8	0.008	0.459	3	remote sensor	19	0.020	0.475	0	flexible sensor	55	0.020	0.471	0
capacitive sensor	8	0.013	0.439	2	strain sensor	18	0.014	0.469	4	remote sensor	52	0.038	0.487	6
immunosensor	8	0.010	0.442	2	glucose sensor	17	0.006	0.440	5	magnetic sensor	51	0.018	0.479	0
potentiometric sensor	8	0.012	0.427	3	humidity sensor	17	0.010	0.456	3	fluorescence sensor	50	0.032	0.458	5
position sensor	7	0.005	0.416	0	amperometric sensor	17	0.010	0.434	3	nanosensor	49	0.019	0.474	3

*Note*: highlighted grey cells indicate emerging units in sensor research after 2000. DC = Degree centrality; BC = Betweenness centrality; CC = closeness centrality.

**Table 3:** Top 20 items in sensor research emerged in networks from 2001 to 2020.

	Top 20 Terms Emerging in Sensor Research			
	2001–2010		2011–2020	
Rank	Label/Item	Degree Centrality	Label/Item	Degree Centrality
1	wireless sensor network	31	self-powered sensor	30
2	wireless sensor	20	environmental sensor	28
3	nano sensor	15	biomedical sensor	22
4	conductometric sensor	11	inductive sensor	21
5	distributed sensor	9	paper sensor	26
6	CMOS sensor	9	low-cost sensor	21
7	CMOS image sensor	9	liquid sensor	19
8	electrochemical biosensor	8	printed sensor	19
9	mass sensor	8	textile sensor	19
10	fiber Bragg grating sensor	8	body sensor network	20
11	refractive index sensor	8	light sensor	18
12	fluorescence sensor	8	mechanical sensor	19
13	active sensor	8	aptasensor	16
14	light-addressable potentiometric sensor	6	dual sensor	16
15	active pixel sensor	6	ratiometric sensor	14
16	colorimetric sensor	6	biomimetic sensor	15
17	flexible sensor	6	chemiresistive sensor	17
18	wearable sensor	6	multifunctional sensor	17
19	DNA sensor	6	visual sensor	13
20	biomimetic sensor	6	copper sensor	13

**Table 4:** Top emerging items associated with sensor technologies in networks from 2001 to 2020.

	Top Emerging Sensor Technologies			
	2001–2010		2011–2020	
Rank	Label/Item	Degree Centrality	Label/Item	Degree Centrality
1	wireless sensor network	31	self-powered sensor	30
2	conductometric sensor	11	biomedical sensor	22
3	distributed sensor	9	inductive sensor	21
4	CMOS image sensor	9	paper sensor	26
5	electrochemical biosensor	8	printed sensor	19
6	fiber Bragg grating sensor	8	textile sensor	19
7	refractive index sensor	8	body sensor network	20
8	fluorescence sensor	8	aptasensor	16
10	light-addressable potentiometric sensor	6	dual sensor	16
11	active pixel sensor	6	ratiometric sensor	14
12	colorimetric sensor	6	biomimetic sensor	15
13	DNA sensor	6	chemiresistive sensor	17
14	biomimetic sensor	6		

## Data Availability

Data Availability at the Web of Science (WOS) 2022. Documents: https://www.webofscience.com/wos/woscc/basic-search (accessed on 15 February 2022).
